# Sexual Practices During Adolescence

**DOI:** 10.1055/s-0040-1713411

**Published:** 2020-11-30

**Authors:** Emanoela Priscila Toledo Arruda, Luiz Gustavo Oliveira Brito, Tatiana Rocha Prandini, Maria Rita Lerri, Rosana Maria dos Reis, Thays Marina Roncato Barcelos, Lúcia Alves Silva Lara

**Affiliations:** 1 Faculdade de Medicina de Ribeirão Preto, Universidade de São Paulo, Ribeirão Preto, SP, Brazil; 2 Universidade Estadual de Campinas, Campinas, SP, Brazil

**Keywords:** sexuality, adolescence, sexarche, condoms, sex education, sexualidade, adolescência, sexarca, preservativos, educação sexual

## Abstract

Adolescence is characterized by significant biological and psychological changes. During this time, the increased production of androgens leads to increased sexual behavior, and this may contribute to early initiation of sexual activity. The objectives of the present cross-sectional study of adolescents enrolled in state schools in the city of Ribeirão Preto, state of São Paulo, Brazil, were to determine the average age at the first sexual intercourse (sexarche), the average number of sexual partners, and the frequency of contraceptive and condom use. Information on the age at sexarche, number of sexual partners, use of different contraceptive methods, and use of condoms were obtained using a semistructured questionnaire. Quantitative variables are expressed as means and standard deviations (SDs), and qualitative variables as absolute and relative frequencies. The chi-squared test was used for comparisons of qualitative variables, and the Student *t* -test for comparisons of continuous variables. All statistical analyses were performed using SAS (version 9.4, North Carolina State University, USA). We evaluated 202 students who answered the questionnaire, 69 males (36.36%) and 133 females (63.64%). The age at sexarche for men ranged from 7 to 18 years old, and for women from 7 to 17 years old. Forty-eight girls (36.01%) and 21 boys (30.43%) were in the first year of high school, 66.94% of adolescents reported sexual intercourse, and 56.25% used a condom during the first sexual intercourse. A total of 36.72% of students said they had safe sex most of the time, and 83.59% said that the first sexual intercourse happened because they “had a crush on” the other person.

## Resumo

A adolescência é caracterizada por significativas mudanças biológicas e psicológicas. Durante esse período, o aumento da produção de andrógenos leva ao aumento do comportamento sexual e isso pode contribuir para o início precoce da atividade sexual. Os objetivos do presente estudo transversal de adolescentes matriculados em escolas estaduais da cidade de Ribeirão Preto, São Paulo, Brasil, foram determinar a idade média na primeira relação sexual (sexarca), o número médio de parceiros sexuais e a frequência de uso de contraceptivos e preservativos. Informações sobre idade na sexarca, número de parceiros sexuais, uso de diferentes métodos contraceptivos e uso de preservativo foram obtidas por meio de um questionário semiestruturado. As variáveis quantitativas são expressas como média e desvio padrão (DP) e as variáveis qualitativas como frequências absolutas e relativas. O teste do qui-quadrado foi usado para comparações de variáveis qualitativas e o teste t de Student para comparações de variáveis contínuas. Todas as análises estatísticas foram realizadas no SAS (version 9.4, North Carolina State University, USA). Foram avaliados 202 estudantes que responderam ao questionário, 69 do sexo masculino (36,36%) e 133 do feminino (63,64%). A idade de sexarca para homens variou de 7 a 18 anos e para mulheres de 7 a 17 anos. Quarenta e oito meninas (36,01%) e 21 meninos (30,43%) cursavam o primeiro ano do ensino médio, 66,94% dos adolescentes relataram relações sexuais e 56,25% usaram camisinha durante a primeira relação sexual. Um total de 36,72% dos estudantes afirmou ter praticado sexo seguro a maior parte do tempo e 83,59% disseram que a primeira relação sexual aconteceu porque “tinham uma queda por” a outra pessoa.

## Introduction

Adolescence is an intermediate stage of human development, between the ages of 10 and 19 years old, 1 that is characterized by remarkable biological and psychological changes. During this phase, an individual first experiences sexuality, a basic need and a central aspect of human beings. 2 Sexuality is intimately related to physical and mental health, and it influences thoughts, feelings, actions, and interactions that are related to sex, gender identity, sexual orientation, eroticism, pleasure, intimacy, and reproduction. 2

The expression of sexuality begins during puberty, when there is increased production of androgens and the development of secondary sexual characteristics. Affective-sexual behavior also increases during this phase, and this can potentiate the emergence of sexual desire and motivate romantic and erotic experiences, 3 leading to increased sexual behavior that manifests as self-eroticism or sexual initiation. These activities are occurring at increasingly younger ages. 4 The World Health Organization (WHO) defines early sexarche as sexual initiation at or before the age of 15 years old. 2

A review of the literature reported that girls benefit when sexarche occurs after they are 16 years old, and that an earlier sexarche adversely affects physical and psychological health. 4 An early sexarche is also associated with unprotected coitus, having more sexual partners, an increased risk of sexually transmitted infections (STIs), and unwanted/unplanned pregnancies. 4 The incidence of HIV continues to increase in certain populations, and has increased in teenagers by 30% during the past 10 years 5 Even though adolescents know the importance of protection against STIs, they still have a low rate of using such protection. Among teenagers living with HIV, more than half reported not using protection against STIs during their last sexual intercourse, and 10% had more than one sexual partner during the previous 12 months. Other STIs are also becoming more common, and HPV infection occurs at an average of 5 months after sexarche. 6

The increased vulnerability of teenagers to pregnancy and STIs is due to numerous factors that affect their sexuality. These factors include impulsivity and egocentric thoughts, 7 young age at sexarche, 8 and lack of information and infrequent or inappropriate use of contraception. 9 10 In fact, a Thai study showed that only ∼ 20% of teenagers used protection against STIs. 11 The PenSe (2015) study of Brazilian teenagers (13 to 17 years old) showed that 27.0% of those who were 13 to 15 years old and 54.7% of those who were 16 to 17 years old were sexually active, and that the older group was significantly more likely to use contraceptive protection (59.7% versus 68.2%). Engaging in risky sexual behavior is also associated with pressure by the partner to initiate the sexual activity, 12 limited education, 13 14 a greater number of sexual partners, 15 16 17 18 and the use of alcohol or drugs.

Many studies have evaluated the behaviors of teenagers in relation with STIs, HIV, and pregnancy, and most have focused on the prevention of pregnancy and STIs. 2 A Brazilian study showed that 89.4% of students in private schools and 87.5% of students in public schools received information about STIs, pregnancy prevention, and how to get free contraceptive protection. 19 Nonetheless, Brazil still has a high rate of pregnancy during adolescence. Adolescence is a good time to build a solid foundation for reproductive health. It must be noted that interventions which aim to reduce STIs and pregnancy in teenagers should not ignore the strong motivations of teenagers to engage in sexual activity. It is possible that curiosity and the search for sexual pleasure are strong motivations to beginning sexual activities during adolescence; however, this theme has received little attention from researchers. The objective of the present study was to assess the motivations of teenagers to initiate sexual activity.

## Methods

**Fig. 1:**
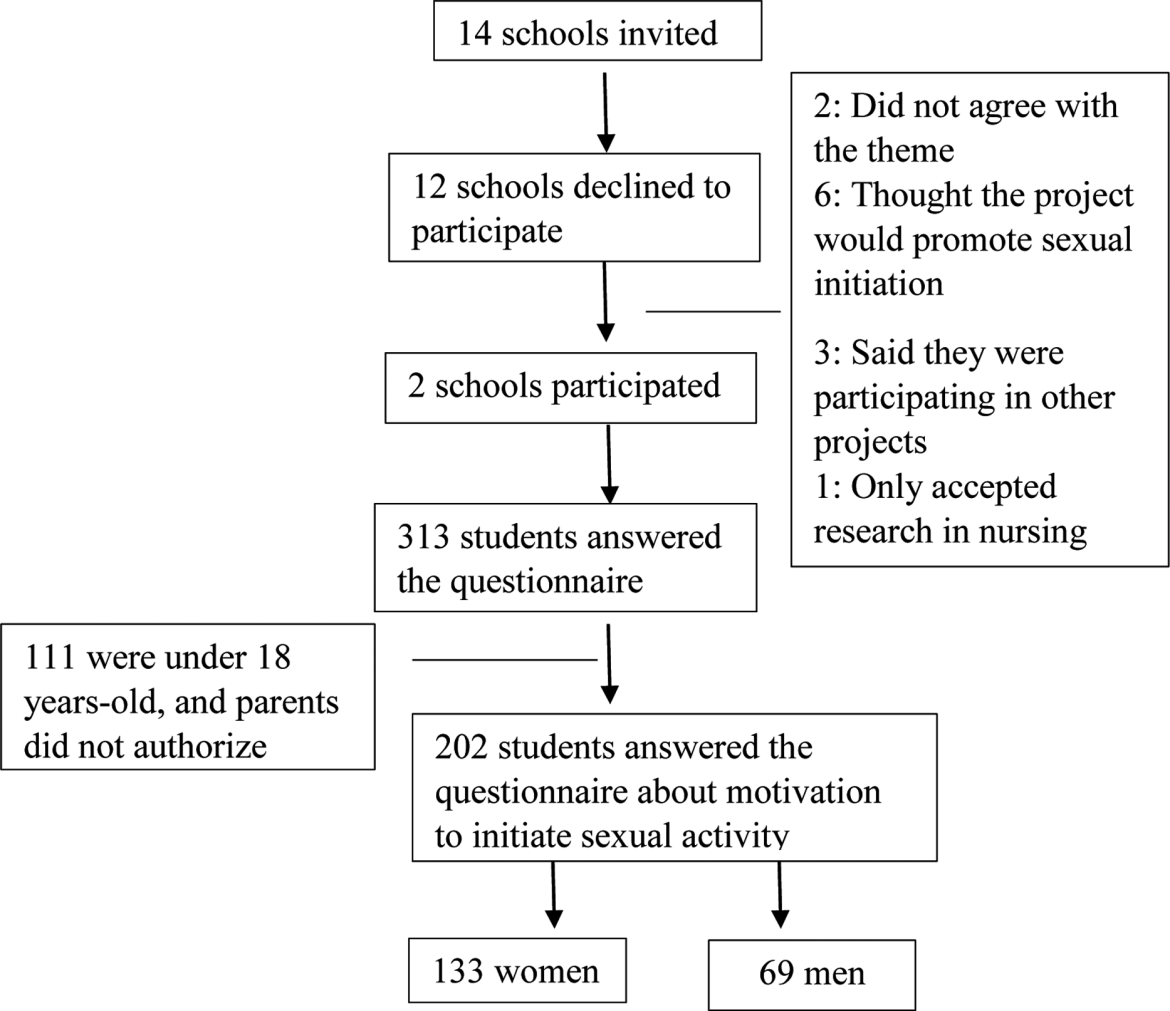
Identification and selection of participating students.

The present cross-sectional study initially considered 14 public high schools in the city of Ribeirão Preto, state of São Paulo, Brazil, that were subjected to random selection from 2016 to 2018. Each school received a number, and using the R program (version 3.2.2, Bell Laboratories, NJ, USA), a random sample of six schools was selected using the sample command procedure. The R program was then used to identify two groups from each of these six schools. Two schools ultimately agreed to participate in the study ( Fig. 1 ). The included students were 15 to 19 years old, and were in the first, second, or third year of high school. Students were excluded if they had cognitive issues that prevented them from understanding the semistructured questionnaire, if they reported embarrassment regarding the questionnaire, or if they said they did not want to participate.

Initially, a meeting was scheduled with the pedagogical coordinator, director, and vice-director during which this project was proposed. Two schools agreed to participate, and they scheduled a meeting with parents to explain the objectives and procedures of the research. These meetings included talking with students and administration of the questionnaires.

The schools sent an Informed Consent Form to the parents of students who were under 18 years old. All the participants or parents/guardians signed the Informed Consent Form. The present research was approved by the Research Ethics Committee of Hospital das Clínicas of the Faculdade de Medicina de Ribeirão Preto.

## Procedures

First, a cycle of lectures was developed with the students using the methodology of a “talk wheel” 20 that had a central theme of human sexuality, sexual function, and sexual initiation. The topics discussed aimed to clarify information about the development of sexuality, and to address issues related to sexual identity, sexual orientation, age at sexarche, safe sexual practices, purpose of the sexual function from the perspective of reproduction and pleasure, the human sexual response, high-risk situations related to sexuality, and the risks and harms from sexual abuse. In all, there were nine talk wheels.

The objective of the first talk wheel was to inform students about the nature and content of the project, and to establish a bond between the students and the psychologist (Arruda E. P. T.) who was responsible for conducting the present research. The psychologist distributed a semistructured questionnaire that asked for information about sociodemographic and clinical characteristics (sex, age, marital status, religion, school grades, socioeconomic status, age at sexarche, motivation for sexual initiation, contraceptive method, number of partners, and use of protection) ( Box 1 ).

**Box 1:** Questionnaire used to collect demographic, clinical, and behavioral data of teenagers

Variable	Answers
Age:	______ years old
Gender:	Female () Male ()
Marital Status	Single () Married () Living together ()
What is your religion?	___________
Do you practice your religion?	Yes () No ()
Housing conditions	Own house () Rent house () Free housing ()
What are your average school grades?	_______
Divorced parents?	Yes () No ()
How many people are living with you?	________ people
Have you ever had sexual intercourse?	Yes () No ()
If you already had sexual intercourse, answer the next questions	
How old were you during your first sexual intercourse?	_______ years old
Did you use protection in your first sexual intercourse?	Yes () No ()
How often do you use protection during sexual intercourse?	()Always () Majority of the times () Rarely () Almost never () Never
What motivated you to have sexual intercourse? You can mark more than one answer	I was in the mood () I wanted to try () I was convinced by my partner () Because my friends already had ()
Questions only to women	
Did you use a contraceptive before the first sexual intercourse?	Yes () No ()
Do you use a contraceptive currently?	Yes () No. Which one? ______________
How many sexual partners have you had?	__________
Do you have a boyfriend/girlfriend?	Yes () No ()

The talk wheels revealed that the teenagers had many questions and doubts regarding sexuality. Based on these talk wheels, the researchers developed a questionnaire with 3 multiple-choice questions to assess the preferences of the students regarding how they would like to receive sexual education ( Box 2 ). This questionnaire was approved by the Ethics Committee. The students responded to this questionnaire in the presence of a teacher and the researcher ((Arruda E. P. T.) on a day defined by the school.

**Box 2:** Semi-structured questionnaire used to evaluate the preferences of students regarding how they would like to receive education about sexuality

Questions	Answers
1. Who would you like to teach you about sexual function/sexuality?	() School () Parents and/or responsible ones () Friends
2. How would you like to receive this information?	() Media () Classes taught by schoolteachers () Specialized lectures () Written documents
3. How would you like to receive information related to your questions about sexuality?	() Pictures () Writing () Writing and pictures

## Statistical Analysis

Data were recorded in an Excel spreadsheet (Microsoft Corporation, Redmond, WA, USA), and then imported into SAS (version 9.4, North Carolina State University, USA). Quantitative variables are reported as means or medians and distributions. Absolute and relative frequencies were used to report qualitative variables. The chi-squared test was used to test the associations of different variables with gender. The Student *t* -test was used to compare the relationship of gender with student age and age at the first sexual intercourse.

## Results

A total of 313 students participated in the talk wheels, and 202 of them provided answers to the initial questionnaire ( Box 1 ) and completed informed consent agreement. There were 69 males (34.2%) and 133 females (65.8%) ( Fig. 1 ).

Among the 202 students, the average age of girls was 16.3 ± 1.0 years old, and of boys it was 16.8 ± 1.0 years old. A total of 128 students (63.4%) said they were sexually active, and 72 of them (56.3%) used protection against STIs during the first sexual intercourse. Forty-seven students (36.7%) reported they had their first sexual intercourse because they were “in the mood.”

When stratified by sex, 68 girls (81%) and 39 boys (88,6%) answered that they were in the mood to initiate the sexual activity, 47 girls (56.0%) and 22 boys (50.0%) reported they wanted to try sexual activity, 8 girls (9.5%) and 5 boys (11.4%) were convinced by their partners to initiate sexual activity, and 5 girls (6.0%) decided to have sexual intercourse because their friends already had had sexual intercourse.

Among girls, the average age at sexarche was 14.6 ± 1.4 years old, 84 (63.4%) reported being sexually active, and the average number of sexual partners was 2.4. Eight girls (6%) reported sexarche at 13 years old, 1 at 12 years old, and 2 at 7 years old.

Among the 44 boys (36.6%), the average age at sexarche was 14.1 ± 2.4 years old, and sexarche occurred in 6 boys (8.7%) at 13 years old, in 3 boys (4.4%) at 12 years old, in 1 boy at 11 years old, in 2 boys 8 years old, and in 1 boy at 7 years old. The boys did not provide answers regarding the number of sexual partners.

Among girls, 41 (60.5%) used protection against STIs during the first sexual intercourse, and 30 (35.7%) continued to use protection during most instances of sexual intercourse. Seventy students (82.4%) reported not using a contraceptive prior to the first sexual intercourse, and 71 (77.2%) reported currently using a contraceptive. A total of 23 boys (52.3%) did not use STI protection during the first sexual intercourse, and 17 boys (38.6%) reported using protection during most instances of sexual intercourse ( Table 1 ).

**Table 1:** Sociodemographic, clinical, and behavioral characteristics of participating teenagers ( *n* = 202)

Variables	*n* (%)	Female *n* (%)	Male *n* (%)
Gender	202 (100)	133 (65.8)	69 (34.2)
Year of high school			
First	69 (34.2)	48 (36.0)	21 (30.4)
Second	66 (32.7)	44 (33.1)	22 (31.9)
Third	67 (33.2)	41 (30.8)	26 (37.7)
Marital status			
Single	192 (95.1)	126 (94.7)	66 (95.7)
Married	3 (1.5)		3 (4.4)
Living together	7 (3.5)	7 (5.3)	
Religion			
Protestant	60 (30.2)		
Catholic	73 (36.7)		
Atheist	3 (1.5)		
None	52 (26.1)		
Others	11 (5.5)		
Religious practice			
Yes	102 (51.0)	76 (57.6)	26 (38.2)
No	98 (49.0)	56 (42.4)	42 (61.8)
Residence			
Own house	143 (70.8)	87 (65.4)	56 (81.2)
Rented house	53 (26.2)	40 (30.1)	13 (18.8)
Free housing	6 (3.0)	6 (4.5)	
Average school grades			
High	40 (19.9)	31 (23.3)	9 (13.2)
Medium	155 (77.1)	99 (74.4)	56 (82.4)
Low	6 (3.0)	3 (2.3)	3 (4.4)
Separated parents			
Yes	102 (50.5)	70 (52.6)	32 (46.4)
No	100 (49.5)	63 (47.4)	37 (53.6)
Already had sexual intercourse			
Yes	128 (63.4)		
No	74 (36.6)		
Used protection during the first sexual intercourse			
Yes	72 (56.3)	51 (60.5)	21 (47.7)
No	56 (43.8)	33 (39.3)	23 (52.3)
Frequency of using protection			
Always	3 (29.7)	24 (28.6)	14 (31.8)
Most of the time	47 (36.7)	30 (35.7)	17 (38.6)
Rarely	16 (12.5)	10 (11.9)	6 (13.6)
Almost never	13 (10.2)	8 (9.5)	5 (11.4)
Never	14 (10.9)	12 (14.3)	2 (4.6)
Motivation at sexarche			
I was in the mood	107 (83.6)	68 (81.0)	39 (88.6)
I wanted to try	69 (53.9)	47 (56.0)	22 (50.0)
I was convinced by a partner	13 (10.2)	8 (9.5)	5 (11.4)
My friends already had sexarche	5 (3.91)	5 (3.91)	0 (0)
Contraceptive and relationships in the teenagers			
Use of contraceptive before the first sexual intercourse			
Yes		15 (17.7)	
No		70 (82.4)	
Do you use a contraceptive currently			
Yes		71 (77.2)	
No		21 (22.8)	
Do you have a boyfriend/girlfriend			
Yes		57 (57.6)	
No		41 (41.4)	

Table 2 shows the results of the semi-structured questionnaire regarding how the teenagers would like to receive sexual education.

**Table 2:** Preferences of teenagers regarding how they would like to receive information about sexuality ( *n* = 313)

Variables	n (%)
Who would you like to teach you about sexual function/sexuality?	
School	122 (39.0)
Parents and/or responsible adults	158 (50.5)
Friends	32 (10.4)
How would you like to receive this information?	
Media	35 (7.01)
Classes taught by schoolteachers	101 (20.4)
Specialized lectures	237 (47.9)
Written documents	122 (24.6)
In what way you most like to communicate questions?	
With pictures	22 (18.01)
With writing	6 (4.9)
With pictures and writing	93 (76.8)

## Discussion

The main objective of the present study was to assess the motivation of teenagers to initiate sexual activity. Notably, only 2 of 14 eligible schools agreed to participate, much less than expected. This suggests that the administrators of public schools are not open to the teaching of sexuality to students. Some of them mentioned the fear that talking about sexuality could increase the sexual activities of teenagers. Instead, age-appropriate education on the broad concepts of sexuality allows health and educational professionals the opportunity to delay the initiation of sexual activity, and reduce risky sexual practices that lead to unplanned/unwanted pregnancies and STIs. 20 21 Education on sexuality using an appropriate methodology is even important for young children, and the combined work of parents, teachers, and health professionals can help to prevent sexual assault and domestic violence. 22

A notable finding of the present study is that ∼ 10% of the teenagers reported sexual initiation when < 13 years old. This suggests vulnerability to sexual violence, because children at this age cannot distinguish sexual abuse from deliberate and consensual sexual relations. Our data thus document a lack of knowledge regarding sexual abuse, and thus reveals the vulnerability of young people to this criminal practice. Previous studies demonstrated that many girls learned about subjects related to puberty from school and family. However, this information is often inaccurate, insufficient, or presented when the girls have already entered puberty. 23

We had access to 202 students (65.84% girls and 34.16% boys) who provided answers to a questionnaire about their reasons for sexual initiation. Among girls, 63% said they had sexual intercourse and the average age at sexarche was 14.6 years old. This would be considered an early sexarche by the World Health Organization (WHO) criteria, 2 and very high rates in relation to European countries. A multicenter study conducted in Austria, Estonia, Germany, Hungary, Ireland, Italy, Romania, Slovenia, and Spain interviewed 10,757 teenagers regarding sexuality. A total of 19.5% reported sexarche when < 15 years old, and 41% reported sexarche when they were > 15 years old. 24

The use of STI protection during the first sexual intercourse in the present study was 60.5% among girls and 47.7% among boys. This result corroborates a previous study in Brazil showing that 77.7% of girls used protection during their most recent sexual experience. 3 It is known that an earlier initiation of sexual activity is associated with a greater likelihood of risky sexual behavior. Thus, interventions that delay the age of the sexarche may be beneficial in preventing risky sexual behaviors.

In the present study, 82.4% of the girls did not use a contraceptive before the first sexual intercourse, indicating a large risk of unwanted/unplanned pregnancies in this population. The rate of pregnancy among adolescents in southeast Brazil is 32%, and 66% of pregnancies during adolescence were not planned in Brazil overall. 25 However, we observed that 77.17% of our population currently used contraceptives. Our results are in line with data from São Paulo, showing that 81.1% of girls used contraceptives. 26 These data are similar to data for American adolescents, in which the use of contraceptives was 74.5% in 2002 and 81.0% in 2011 to 2015, 27 thus indicating an increasing tendency for girls to use a contraceptive after the sexarche. The implementation of methods that increase the proper use of a contraceptive before the first sexual intercourse is urgent.

Our evaluation of the motivation for sexual initiation indicated that 81% of girls and 89% of boys had the first sexual intercourse because they were “in the mood.” It must be noted that increased interest in sex occurs because hormonal changes during the pubertal period increase the search for sexual sensations, 28 and this can trigger increased sexual activities in young people. Another motivation for sexual initiation in more than half of the girls and boys was their curiosity in wanting to “try” sexual intercourse. This result is in agreement with data from São Paulo reporting the main reasons for initiating the first sexual intercourse were attraction, curiosity, and the wish to not be a virgin. 29 Another motivation for sexual initiation (9.5% of girls and 11.4% of boys) was pressure from the partner to have sexual intercourse; in addition, 6% of girls were influenced by friends who already had had sexual intercourse. These findings agree with those of a study in São Paulo, in which 86.2% of teenagers reported that the first sexual intercourse was desired, 12.5% reported that the first intercourse was consensual, but not wanted, and 1.3% reported that the first sexual intercourse was rape. 26

Knowledge of why teenagers want to initiate sexual activity may help to develop interventions for this population. In particular, educational interventions may help them to better deal with their increasing sexual desires, individual and shared sexual sensations, and therefore reduce the risk of unprotected sexual intercourse, STIs, and pregnancy. It is important to highlight that boys feel empowered by the experience of sexual initiation because it affirms their masculinity. 30 Thus, they may insist to have sex and girls may give in to the pressure to have sex by initiating sex without adequate protection.

The negative repercussions of young age at sexarche are well known and include engaging in risky sexual behaviors, use of recreational drugs, victimization, and suicidal thoughts. 4 Violence against women and infection by HIV are also associated with sexual initiation before the age of 15 years old. 31 Thus, a young age at sexarche is associated with many adverse events, so this problem must be addressed with public policies that aim to provide sexual education and improve sexual health.

Our analysis of the preferences of adolescents for sources of information about sexual education indicated that 50.5% of them wanted their parents and/or guardians to guide them, 47.9% wanted to receive information through specialized lectures, and 76.8% wanted to receive written and illustrated materials. These findings are the first to identify how adolescents want to be educated about sexuality. These important data are essential for the development of programs that aim to promote education in sexuality, and indicated that parents included during school meetings may help children to develop a healthy sexuality. The question “How would you like to receive information related to your questions about sexuality?” gave rise to a paper entitled “What do teenagers want to know about sexuality?” The results of this work were published elsewhere. 32 This publication provides adults with information about the questions that adolescents have about sexuality. This material may help to guide the design of sexual education programs in schools, based on the needs and desires of the adolescents themselves. It is necessary to conduct a randomized study to compare methods currently used with a new protocol that considers the questions and needs of adolescents.

The main limitation of the present study was the examination of a small number of schools, and this limits the generalizability of our findings. Additionally, we did not use validated questionnaires, because there are no available validated questionnaires in Brazil for assessment of the variables of interest. However, the “talk wheels” created an opportunity to raise important issues about the preferences of students regarding getting in touch with the theme of sexuality. Based on our data, better protocols can be developed for sexual education in schools that are based on the self-reported needs of the students themselves.

The three questions we used to assess the preferences of teenagers regarding how they would like to receive information about sexuality ( Box 2 ) strengthened our study. This is unique, because no previous study has examined this issue. We believe that programs designed based on the expressed concerns of adolescents may be more effective than those designed simply based on academic publications. Sex education in schools has the potential to promote the formation of self-awareness and values in children and adolescents so that they make better decisions and more appropriate choices. Sexuality is an intrinsic aspect of human beings during all stages of life, and sex education in schools, conducted in partnership with parents and/or guardians, can provide adolescents with critical information that counteracts the bombardment of harmful or distorted information from the media and elsewhere. According to the United Nations Educational, Scientific and Cultural Organization (UNESCO), children and adolescents are often exposed to misinformation about sexuality, rather than systematic, evidence-based sex education. 26

Finally, our data suggest that the high school period provides a window of opportunity to parents and teachers, who can educate teenagers regarding healthy sexual experiences. Also, children need be taught to protect themselves from sexual violence, and programs developed and administered by doctors, teachers, and psychologists should focus on this theme. Schools can provide windows of opportunity that teachers and parents together can use to provide age-appropriate education that reduces the risk of sexual violence against children and teenagers. Our results indicated that adolescents do not understand that the first sexual intercourse must be a consensual act, and that a child does not have the maturity to provide consent to any sexual activities with adults. The lack of such education in Brazil leads to teenagers initiating their sexual life unprepared and vulnerable to unwanted pregnancy and STIs. Health and education professionals, supported by the government, should develop sexual education programs that guarantee that teenagers have healthy sexual experiences.

## Conclusion

More than half of the students in the public schools of Ribeirão Preto had early sexual initiation. Most of these teenagers initiated sexual intercourse without protection from STIs or contraception. Most of the students reported preferring to receive information about sexuality from teachers and parents, and most preferred to receive this information from specialized lectures. “Being in the mood” and wanting to “experience sexual intercourse” were the main motivations for sexual initiation. We believe the results of the present study, which identified the preferences of teenagers regarding how they would like to be educated about sexuality, can contribute to the development and elaboration of programs that provide appropriate sexual education to teenagers in schools. This approach can reduce risky sexual behaviors and generally improve the sexual health of teenagers.
